# Association of ADHD and LD with vision abnormalities among the children and adolescents in US, NHANES 1999–2004

**DOI:** 10.3389/fneur.2025.1465444

**Published:** 2025-02-24

**Authors:** Jiamin Lu, Kefan Zhou, Yingyu Liang, Qishan Li, Jiawen Zhong, Xia Zeng, Tianran Shen, Chongzhen Sun, Xinping Yu, Jinhua Lu, Wenhan Yang

**Affiliations:** ^1^Department of Child and Adolescent Health, School of Public Health, Guangdong Pharmaceutical University, Guangzhou, China; ^2^Department of Nutrition and Food Health, School of Public Health, Guangdong Pharmaceutical University, Guangzhou, China; ^3^State Key Laboratory of Ophthalmology, Zhongshan Ophthalmic Center, Sun Yat-sen University, Guangdong Provincial Key Laboratory of Ophthalmology and Visual Science, Guangdong Provincial Clinical Research Center for Ocular Diseases, Guangzhou, China; ^4^Division of Birth Cohort Study, Guangzhou Women and Children’s Medical Center, Guangzhou Medical University, Guangzhou, China

**Keywords:** refractive error, attention-deficit/hyperactivity disorder, ADHD, learning disabilities, vision

## Abstract

**Objective:**

To examine the association of ADHD and LD with visual impairment, uncorrected refractive error, and refractive error (myopia, hyperopia, and astigmatism) among US children and adolescents.

**Method:**

A population-based cross-sectional study included 3,385 participants aged 12–15 years from the large, representative sample of US NHANES. The diagnoses of ADHD and LD in children and adolescents, as reported by parents or adolescents themselves, were analyzed. All participants’ right eyes were used to calculate the spherical equivalent refractive errors (SER) during the investigation. Myopia, hyperopia, and astigmatism were classified by SER. Visual acuity was categorized into normal, uncorrected refractive error (URE), and visual impairment (VI) according to objectively assessed for each eye. Logistic regression analysis was used to estimate the associations between ADHD and LD and vision abnormalities.

**Results:**

Among a total of 3,385 children and adolescents aged 12–15 years, 279 were reported to have a diagnosis of ADHD, and 427 were reported to have a diagnosis of LD. Compared with those without ADHD, children and adolescents with ADHD had an increased risk of hyperopia, with odds ratios (ORs) of 1.66 (95% CI, 1.03–2.67). LD was associated with higher risks of hyperopia (OR = 1.85, 95% CI, 1.17–2.90) and astigmatism (OR = 1.63, 95% CI: 1.18–2.26). After controlling for confounding variables, the results remained stable. LD also increased the risk of vision impairment (OR = 3.05, 95% CI: 1.05–8.90) after controlling for confounders. Stratified analyses showed that ADHD was a risk factor for hyperopia in boys compared with girls (OR = 1.62, 95%CI = 1.03–2.72), in 12–13-year-old individuals compared with 14–15-year-olds (OR = 1.69, 95%CI = 1.05–3.42). LD was a risk factor for hyperopia and astigmatism in girls compared with boys (OR = 2.81, 95%CI = 1.53–5.14; OR = 2.18, 95%CI = 1.22–3.90), and in 12–13-year-old individuals compared with 14–15-year-olds (OR = 1.99, 95%CI = 1.16–3.42; OR = 1.65, 95%CI = 1.07–2.56).

**Conclusion:**

Children and adolescents diagnosed with ADHD and LD may be at a greater risk of developing hyperopia and astigmatism. To accurately diagnose and treat children affected by ADHD and LD, healthcare practitioners from various medical specialties should take this association into account.

## 1 Introduction

Vision has wide-ranging and significant effects on the economy, sustainable development, health, and many other aspects of life. Vision abnormalities, including refractive errors (myopia, hyperopia, astigmatism), strabismus, uncorrected refractive error, and vision impairment, can cause reading difficulties, blindness, anxiety, anti-social behavior, and problems with quality of life (1, 2). An estimated 596 million individuals worldwide suffered from distance vision impairment in 2020, 43 million of whom were blind. A further 510 million people lacked reading glasses, which resulted in uncorrected near vision impairment (3). It was anticipated that the number of people with myopia and high myopia would have significantly increased worldwide. Not only this, but hyperopia and other vision problems should also be of concern. Refractive errors are common in children, which suggests that they are a particularly vulnerable population (4). The prevalence of uncorrected refractive errors and strabismus in children ranged from 7.7 to 10.3% (5, 6), and uncorrected refractive problems cause vision impairment in about two-thirds of children worldwide (7).

Vision is the main sensory modality through which human beings acquire information, and Treichler stated that 80% of an individual’s information comes from vision (8). After light strikes the retina in the back of our eyes, the visual information is transmitted to the primary visual cortex (V1) and dorsal lateral geniculate nuclei (LGN), which together form the image-forming visual circuit. Among these, dorsal and ventral pathways are involved in visual perception and visuo-motor coordination (9, 10). Vision is critical in the development of movement and cognition and influences all skills learned in early life. Through the brain’s processing of visual information, we are able to identify and perceive the world around us, including colors, shapes, contours, and motion. Also, vision guides and controls our behavior. Our behavior is based on our perception and understanding of our environment. In infants, it promotes the development of early motor functions such as head control, facial expressions, imitation, and grasping objects (11, 12).

Children’s behavior and development, especially their attentional skills, learning, and reading processes, are influenced by their visual perception (13). Refractive errors can lead to deficits in these abilities and their development. Refractive error studies have shown that children with hyperopia perform worse on visual cognition and visuomotor tasks compared to children without refractive error. Children with hyperopia performed worse on reading and writing examinations as well as on standardized assessment tests in science, math, and English (14).

Attention deficit/hyperactivity disorder (ADHD) and Learning disabilities (LD) are the most frequently encountered neurodevelopmental disorders in childhood. Attention-deficit hyperactivity disorder (ADHD) is characterized by a recurrent pattern of inattention, hyperactivity, and impulsivity (15), affecting 5% of children and adolescents and 3% of adults (16, 17). Learning disabilities can affect neurocognitive processes and may limit the capacity to read, listen, spell, speak, write, concentrate, or organize information (18). The most prevalent form of learning disability is dyslexia, or a reading disability (19). ADHD and LD are neurodevelopmental disorders that manifest as structural abnormalities in the brain (20), and the same embryological tissue from which the brain develops also gives rise to the structures of the eye (21).

Some studies have elucidated the link between visual problems and ADHD/LD. There is recent meta-analytic evidence of disorders of the eye, such as astigmatism, hyperopia, strabismus, and altered measures of visual function, in people with ADHD compared to those without ADHD (22). The complex relationship between vision and ADHD/LD was further demonstrated by early abnormalities in visual sensory integration utilizing event-related potentials assessed in the visual cortex of children with ADHD (23), and the people with ADHD processed visual stimuli more slowly. Moreover, a literature review of the visual search performance of children with ADHD reported that these children were significantly slower on a sequential search task compared to their normally developing peers (24). People with nonverbal learning disabilities were impaired in visuospatial processing speed, visual perceptual ability, visual construction ability, and visuospatial working memory (25). In addition, although it is widely accepted that dyslexia is due to deficits in phonological and verbal information processing (26, 27), a growing body of data suggests that visual attention deficits contribute to reading difficulties in children with dyslexia. Therefore, it is reasonable to hypothesize that visual cognition abnormalities are more likely to be comorbid with ADHD or LD, potentially stemming from shared neurobiological factors.

After reviewing the evidence regarding visual anomalies and LD or ADHD, we identified the following limitations. Only a few studies have investigated the relationship between visual anomalies and LD or ADHD, and the findings have been inconsistent. For example, while some studies have indicated an increase in the prevalence of vision issues, including amblyopia, strabismus, hyperopia, astigmatism, heterotopia (28, 29), altered color vision, and contrast sensitivity in individuals with ADHD, another study found no association (30). Currently, there is insufficient scientific evidence to support the view that children with ADHD and LD have more visual problems. Therefore, in this study, based on the nationally representative sample in US, we aimed to investigate the prevalence of visual problems in children and adolescents with and without ADHD or LD, and to examine the association between ADHD and LD and visual abnormalities in adolescents.

## 2 Methods

### 2.1 Study population

The data used in this study were derived from the merger of three cycles (1999–2004) of the National Health and Nutrition Examination Survey (NHANES). NHANES is a nationally representative, cross-sectional sample obtained through a stratified, multistage probability design. It was routinely conducted by the Centers for Disease Control and Prevention of the non-institutionalized civilian populations in the United States (31).

Initially, the present analysis involved a total of 3,393 participants with and without ADHD and LD aged 12–15 years with valid information regarding vision examination data. Participants with a history of previous refractive surgery (4), history of cataract surgery (1) and corneal disease (3), as indicated by keratometry readings of greater than 50 D (32, 33), were excluded. Consequently, 3,385 adolescents with and without ADHD and LD were ultimately included for the primary analysis.

### 2.2 Measurements and variable

#### 2.2.1 Refractive errors

Objective refraction data was obtained by the auto-refractor/keratometer (model ARK-760A) (34). In order to facilitate the analysis, spherical equivalent refractive errors, which are the average of the refractions in the two principal meridians, were calculated from the data of the right eyes of all participants (35). Myopia was defined as a SER equal to or less than-0.75 D, this more cautious definition of myopia was selected to avoid misclassification of myopia because cycloplegic medications were not used to detect refractive abnormalities. Hyperopia as a SER equal to or greater than +0.5 D, and astigmatism as a cylinder equal to or greater than 1.0 DC.

#### 2.2.2 Visual acuity

Presenting visual acuity was assessed, and participants were asked to wear any regular distance vision correction. The 20/50 line was introduced first. The 20/200 line was shown if the participant was unable to read the 20/50 line. Participants’ visual acuity was rated as being worse than 20/200 if they were unable to read the 20/200 line. Participants were permitted to continue to the next line of smaller characters as long as they could properly read 4 or 5 items for the 20/50 line. This continued until the contestant missed two consecutive lines of two or more characters each or read the 20/20 line correctly. The last line for which four or more characters were read correctly was recorded as representing present visual acuity. It was not evaluated for visual acuity.

Corrected lenses were taken out after measuring the presenter’s visual acuity, and the autorefractor was used to determine each eye’s refraction. The measured refractive error correction was used to calculate the corrected visual acuity for eyes with a presenting visual acuity of less than 20/25. The better-seeing eye’s visual acuity was employed to describe visual impairment status.

Individuals with a presenting visual acuity of 20/40 or better were considered to have normal vision. URE was defined as those whose postrefraction visual acuity was 20/40 or greater but whose presenting visual acuity was less than 20/40. Individuals classified as VI were those whose visual acuity remained below 20/40 even after autorefraction.

#### 2.2.3 Measurement of diagnosed LD and ADHD

Our primary outcome variables, LD and ADHD, were based on self-report/guardian responses to two NHANES interview questions: “Has a representative from a school or a health professional ever told (the child) that (he/she) had a learning disability?” and “Has a doctor or health professional ever told (the child) that (he/she) had attention deficit disorder?” The identical ADHD questions were asked of kids who were 16 years old or older, but the terms in parenthesis were changed to (you) and (you), respectively.

#### 2.2.4 Assessment of covariates

Questionnaires were used to gather data on participant gender, age, race/ethnicity, household income, and BMI. Non-Hispanic white, non-Hispanic black, Mexican-American, and other races/ethnicities were the categories used to classify race and ethnicity. The 2,000 Centers for Disease Control Growth Charts were used as the basis for a computerized algorithm that estimated BMI in kilograms per square meter. The result was a conversion to BMI percentile values that were particular to age and sex (36). Each individual was placed in one of three BMI strata: obesity (95th percentile), overweight (85th to 94th percentile), or normal (85th percentile).

### 2.3 Data analyses

The NHANES analytical guidelines for 1999–2004 are available at www.cdc.gov/nchs/data/series/sr_02/sr02_161.pd. The survey modules of SAS software, version 9.4, were used to compute descriptive analysis and concurrent and prospective associations. For continuous variables, ANOVA was used to evaluate the means and proportions of the baseline characteristics; for categorical variables, chi-square testing was used. We used binary logistic regression to estimate the association between refractive error (myopia, hyperopia, astigmatism), visual acuity, and ADHD or LD. Model 1 adjusted for adolescent sex and age. Model 2 adjusted for age, sex, race/ethnicity, household income level, and BMI. In addition, we ran a stratified analysis to examine whether this association differed by sex, age, and ethnicity. Statistical significance was evaluated two-sided *p*-value at the 5% level.

## 3 Results

### 3.1 Characteristics of participants with ADHD and LD

The characteristics of participants with ADHD and LD are described in Table 1. The study population of 3,385 adolescents aged 12–15 years comprised 279 adolescents with ADHD (8.24%) and 3,106 adolescents without ADHD (91.76%), and 427 adolescents with LD (12.61%) and 2,958 adolescents without LD (87.19%), with a weighted mean (SE) age of 13.46 (0.02) years; 1,660 boys (weighted, 51.38%) and 1725 girls (weighted, 48.62%). Within the ADHD group, 15.37% (*N* = 66) children showed hyperopia, while 10.97% (*N* = 623) of the control group had hyperopia (χ2 = 4.16; *p* < 0.05). But, we found no differences observed for myopia, hyperopia, astigmatism, visual acuity, and ADHD. Within the LD group, 18.70% (*N* = 66) children showed hyperopia, while 11.08% (*N* = 305) of the control group had hyperopia (χ2 = 5.17; *p* = 0.023). Furthermore, significant group differences appeared between the LD group and the control group regarding astigmatism. Over 21% (*N* = 86) of the LD and 14.18% (*N* = 465) of the control group suffered from astigmatism (χ2 = 7.98; *p* = 0.005). But, we found no significant differences were observed for myopia, visual acuity and LD.

**Table 1:** Characteristics of overall participants aged 12–15 years according to ADHD and LD (*N* = 3,385) in NHANES, 1999-2004.^a^

Variables	Total	ADHD		*χ* ^***2***^ */F*	*P*-value	LD		*χ* ^***2***^ */F*	*P-*value
		Yes	No			Yes	No		
No. of participants	3,385	279	3,106			427	2,958		
Age, y [Mean (SE)]	13.46(0.02)	13.30 (0.07)	13.49 (0.02)	5.87	0.020	13.45 (0.06)	13.48 (0.02)	0.09	0.763
Sex, *N* (%)									
Male	1,660 (51.38)	205 (74.72)	1,455 (48.20)	52.59	<0.001	269 (66.24)	1,391 (48.91)	24.46	<0.001
Female	1725 (48.62)	74 (25.28)	1,651 (51.80)			158 (33.76)	1,567 (51.10)		
Race/ethnicity, *N* (%)									
Hispanic	1,325 (17.98)	58 (8.54)	1,267 (19.27)	16.66	<0.001	139 (13.89)	1,186 (18.66)	5.50	0.139
Non-Hispanic White	864 (61.38)	118 (74.97)	746 (59.53)			133 (67.82)	731 (60.31)		
Non-Hispanic Black	1,074 (14.63)	90 (10.43)	984 (15.20)			143 (13.76)	931 (14.77)		
Other	122 (6.01)	13 (6.06)	109 (6.00)			12 (4.52)	110 (6.26)		
Family income to poverty ratio, *N* (%)									
<=1.30	1,320 (28.89)	94 (26.40)	1,226 (29.23)	6.05	0.109	196 (35.16)	1,124 (27.85)	6.92	0.074
1.31–3.50	1,141 (35.09)	110 (43.07)	1,031 (34.00)			141 (35.94)	1,000 (34.93)		
>3.5	670 (30.22)	63 (26.69)	607 (30.69)			64 (22.54)	606 (31.49)		
Missing	254 (5.81)	12 (3.83)	242 (6.08)			26 (6.36)	228 (5.72)		
BMI, kg/m2, N(%)									
Below	65 (2.74)	11 (7.57)	54 (2.08)	12.84	0.005	13 (5.41)	52 (2.29)	8.97	0.030
Normal	1980 (61.78)	166 (59.82)	1814 (62.04)			230 (57.59)	1750 (62.47)		
Overweight	617 (17.39)	46 (17.47)	571 (17.38)			73 (13.39)	544 (18.06)		
Obesity	723 (18.09)	56 (15.19)	667 (18.49)			111 (23.60)	612 (17.17)		
Cycle, *N* (%)									
1999–2000	1,151 (29.15)	83 (28.76)	1,068 (29.20)	0.41	0.814	142 (30.53)	1,009 (28.92)	1.24	0.537
2001–2002	1,174 (36.67)	90 (34.40)	1,084 (36.97)			153 (38.88)	1,021 (36.29)		
2003–2004	1,060 (34.19)	106 (36.84)	954 (33.83)			132 (30.59)	928 (34.79)		
Refractive status, *N* (%)									
No myopia	2,286 (67.85)	197 (68.87)	2089 (67.71)	0.15	0.698	308 (70.63)	1978 (67.38)	0.89	0.347
myopia^b^	1,099 (32.15)	82 (31.13)	1,017 (32.29)			119 (29.37)	980 (32.62)		
No hyperopia	3,014 (87.83)	238 (82.34)	2,776 (88.58)	3.15	0.076	361 (81.30)	2,653 (88.92)	5.17	0.023
Hyperopia^c^	371 (12.17)	41 (17.66)	330 (11.42)			66 (18.70)	305 (11.08)		
No astigmatism	2,834 (84.81)	233 (83.95)	2,601 (84.93)	0.13	0.723	341 (78.75)	2,493 (85.82)	7.98	0.005
astigmatism^d^	551 (15.19)	46 (16.05)	505 (15.07)			86 (21.25)	465 (14.18)		
Visual acuity, *N* (%)									
Normal^e^	2,960 (89.66)	239 (86.70)	2,721 (90.06)	1.85	0.397	366 (88.02)	2,594 (89.93)	2.40	0.301
URE^f^	393 (9.52)	36 (12.03)	357 (9.18)			54 (10.21)	339 (9.41)		
VI^g^	32 (0.82)	4 (1.28)	28 (0.76)			7 (1.77)	25 (0.66)		

NHANES, National Health and Nutrition Examination Survey.

^aFor continuous variables, the data are shown as weighted means and standard errors in parentheses; for categorical variables, the data are provided as frequencies and weighted percentages in parenthesis.^

^bSpherical Equivalent Refraction equal to or less than-0.75 DS.^

^c^Spherical Equivalent Refraction equal to or greater tha*n* + 0.5 DS.

^dCylinder equal to or greater than 1.0 DC.^

^ePresenting visual acuity equal to or better than 20/40.^

^fUncorrected refractive error, presenting visual acuity worse than 20/40 but improving to 20/40 or better with autorefraction.^

^gVisual impairment, visual acuity worse than 20/40 even after autorefraction.^

### 3.2 Association of vision abnormalities with ADHD and LD

The odds ratios (ORs) of myopia, hyperopia, and astigmatism with ADHD and LD in adolescents are listed in Table 2. Within the ADHD group, in the crude model, children and adolescents with ADHD had a risk for the prevalence of hyperopia with odds ratio (ORs) of 1.66 (95% CI, 1.03–2.67) compared with those without ADHD. After adjustment for age and sex, the odds ratio of ADHD associated with hyperopia was 1.66 (95% CI, 1.05–2.61). In Model 2, which was additionally adjusted for age, sex, race/ethnicity, poverty-income ratio, and body mass index (BMI), the ORs and 95% CIs remained stable.

**Table 2:** The association between and ADHD and LD and vision problem in NHANES, 1999–2004.

Outcomes	ADHD, OR (95% CI)		*P*-value	LD, OR (95% CI)		*P*-value
	No	Yes		No	Yes	
Myopia						
Case/Total	1017/3106	82/279		980/2958	119/427	
Crude model	1.00 (Reference)	0.95 (0.72, 1.25)	0.702	1.00 (Reference)	0.86 (0.62, 1.20)	0.363
Model 1	1.00 (Reference)	0.99 (0.73, 1.34)	0.923	1.00 (Reference)	0.87 (0.62, 1.22)	0.414
Model 2	1.00 (Reference)	1.02 (0.73, 1.41)	0.908	1.00 (Reference)	0.88 (0.63, 1.23)	0.452
Hyperopia						
Case/Total	330/3106	41/279		305/2958	66/427	
Crude model	1.00 (Reference)	1.66 (1.03, 2.67)	0.036	1.00 (Reference)	1.85 (1.17, 2.90)	0.009
Model 1	1.00 (Reference)	1.66 (1.05, 2.61)	0.030	1.00 (Reference)	1.83 (1.17, 2.86)	0.010
Model 2	1.00 (Reference)	1.60 (1.04, 2.46)	0.035	1.00 (Reference)	1.77 (1.15, 2.75)	0.011
Astigmatism						
Case/Total	505/3106	46/279		465/2958	86/427	
Crude model	1.00 (Reference)	1.07 (0.72, 1.62)	0.717	1.00 (Reference)	1.63 (1.18, 2.26)	0.004
Model 1	1.00 (Reference)	1.03 (0.70, 1.52)	0.892	1.00 (Reference)	1.58 (1.13, 2.21)	0.009
Model 2	1.00 (Reference)	1.08 (0.69, 1.69)	0.735	1.00 (Reference)	1.54 (1.11, 2.13)	0.010
Visual acuity						
URE						
Case/Total	357/3106	36/279		339/2958	54/427	
Crude model	1.00 (Reference)	1.36 (0.89, 2.09)	0.154	1.00 (Reference)	1.11 (0.73, 1.69)	0.622
Model 1	1.00 (Reference)	1.42 (0.91, 2.21)	0.116	1.00 (Reference)	1.14 (0.75, 1.75)	0.531
Model 2	1.00 (Reference)	1.51 (0.98, 2.32)	0.062	1.00 (Reference)	1.15 (0.79, 1.69)	0.461
VI						
Case/Total	28/3106	4/279		25/2958	7/427	
Crude model	1.00 (Reference)	1.75 (0.41,7.48)	0.440	1.00 (Reference)	2.74 (0.83,9.07)	0.097
Model 1	1.00 (Reference)	1.96 (0.49,7.78)	0.331	1.00 (Reference)	3.05 (1.05,8.90)	0.042
Model 2	1.00 (Reference)	1.80 (0.42,7.78)	0.425	1.00 (Reference)	2.60 (0.84,8.03)	0.096

NHANES, National Health and Nutrition Examination Survey; OR, odds ratio; URE, uncorrected refractive error; VI, vision impairment.

Crude model adjusted for none.

Model 1 adjusted for age and sex.

Model 2 adjusted age, sex, race/ethnicity, ratio of family income to federal poverty threshold and BMI.

Within the LD group, in the crude model, children and adolescents with LD had a risk for the prevalence of hyperopia with odds ratios (ORs) of 1.85 (95% CI, 1.17–2.90) and astigmatism with odds ratios (ORs) of 1.63 (95% CI, 1.18–2.26) compared with those without LD. After adjustment for age and sex, results indicated a higher risk for prevalence of hyperopia (OR = 1.83; 95% CI, 1.17–2.86) and astigmatism (OR = 1.58, 95% CI, 1.13–2.21) in children with LD. In Model 2, which was additionally adjusted for potential confounding variables (age, sex, race/ethnicity, poverty-income ratio, and BMI), the ORs and 95% CIs remained stable. Moreover, there was a significantly association between LD and vision impairmen (OR = 3.05, 95% CI:1.05–8.90) after controlling for confounding variables. However, no significant associations were observed between children and adolescents with ADHD and myopia, uncorrected refractive error and vision impairment, adjusted ORs remained nonsignificant after controlling for other confounding variables. Similarly, no significant associations were observed between LD and myopia and uncorrected refractive error.

### 3.3 Stratified analysis

Stratified analyses showed that ADHD was a risk factor for hyperopia in boys compared girls (OR = 1.62, 95%CI = 1.03–2.72), in 12–13-year-old individuals compared with 14–15-year-olds (OR = 1.69, 95%CI = 1.05–3.42), and in Non-Hispanic White/Others compared with Hispanic and Non-Hispanic Black (OR = 1.74, 95%CI = 1.05–2.91). Having LD was a risk factor for prevalence of hyperopia and astigmatism in girls compared with boys (OR = 2.81, 95%CI = 1.53–5.14; OR = 2.18, 95%CI = 1.22–3.90), and in 12–13-year-old individuals compared with 14–15-year-olds (OR = 1.99, 95%CI = 1.16–3.42; OR = 1.65, 95%CI = 1.07–2.56) (Table 3).

**Table 3:** Stratified analyses of the association of ADHD and LD with vision abnormalities in US adolescents aged 12–15 years in NHANES, 1999–2004.

Outcomes	Without ADHD		ADHD		Without LD		LD	
	Case/Total	OR (95%CI)	Case/Total	OR (95%CI)	Case/Total	OR (95%CI)	Case/Total	OR (95%CI)
Myopia								
Sex								
Male	464/1455	1.00 (Reference)	59/205	0.96 (0.68, 1.36)	446/1391	1.00 (Reference)	77/269	1.01 (0.66, 1.53)
Female	553/1651	1.00 (Reference)	23/74	1.02 (0.50, 2.07)	534/1567	1.00 (Reference)	42/158	0.68 (0.39, 1.19)
Age								
12–13	492/1587	1.00 (Reference)	48/160	1.22 (0.74, 2.01)	472/1519	1.00 (Reference)	68/228	0.97 (0.58, 1.63)
14–15	525/1519	1.00 (Reference)	34/119	0.75 (0.50, 1.10)	508/1439	1.00 (Reference)	51/199	0.78 (0.55, 1.09)
Race/ethnicity								
Hispanic	427/1267	1.00 (Reference)	19/58	1.58 (0.78, 3.19)	408/1186	1.00 (Reference)	38/139	1.32 (0.76, 2.31)
Non-Hispanic White/Others	268/855	1.00 (Reference)	38/131	0.92 (0.64, 1.31)	265/841	1.00 (Reference)	41/145	0.76 (0.54, 1.09)
Non-Hispanic Black	322/984	1.00 (Reference)	25/90	0.79 (0.49, 1.26)	307/931	1.00 (Reference)	40/143	0.80 (0.47, 1.38)
Hyperopia								
Sex								
Male	145/1455	1.00 (Reference)	28/205	1.62 (1.03, 2.56)^a^	140/1391	1.00 (Reference)	33/269	1.27 (0.69, 2.38)
Female	185/1651	1.00 (Reference)	13/74	1.40 (0.66, 2.97)	165/1567	1.00 (Reference)	33/158	2.81 (1.53, 5.14)^a^
Age								
12–13	180/1587	1.00 (Reference)	24/160	1.69 (1.05, 2.72)^a^	169/1519	1.00 (Reference)	35/228	1.99 (1.16, 3.42)^a^
14-15	150/1519	1.00 (Reference)	17/119	1.40 (0.65, 3.00)	136/1439	1.00 (Reference)	31/199	1.55 (0.80, 2.99)
Race/ethnicity								
Hispanic	119/1267	1.00 (Reference)	4/58	0.45 (0.15, 1.39)	107/1186	1.00 (Reference)	16/139	1.04 (0.48, 2.24)
Non-Hispanic White/Others	101/855	1.00 (Reference)	24/131	1.74 (1.05, 2.91)^a^	96/841	1.00 (Reference)	29/145	1.97 (1.15, 3.37)^a^
Non-Hispanic Black	110/984	1.00 (Reference)	13/90	1.54 (0.77, 3.09)	102/931	1.00 (Reference)	21/143	1.60 (1.03, 2.48)^a^
Astigmatism								
Sex								
Male	268/1455	1.00 (Reference)	35/205	1.23 (0.68, 2.23)	250/1391	1.00 (Reference)	53/269	1.21 (0.84, 1.74)
Female	237/1651	1.00 (Reference)	11/74	0.67 (0.28, 1.61)	215/1567	1.00 (Reference)	33/158	2.18 (1.22, 3.90)^a^
Age								
12–13	245/1587	1.00 (Reference)	28/160	1.51 (0.77, 2.94)	230/1519	1.00 (Reference)	43/228	1.65 (1.07, 2.56)^a^
14-15	260/1519	1.00 (Reference)	18/119	0.77 (0.43, 1.36)	235/1439	1.00 (Reference)	43/199	1.49 (0.92, 2.42)
Race/ethnicity								
Hispanic	189/1267	1.00 (Reference)	8/58	0.58 (0.26, 1.32)	175/1186	1.00 (Reference)	22/139	1.33 (0.70, 2.54)
Non-Hispanic White/Others	124/855	1.00 (Reference)	22/131	1.13 (0.66, 1.94)	111/841	1.00 (Reference)	35/145	1.63 (1.03, 2.59)^a^
Non-Hispanic Black	192/984	1.00 (Reference)	16/90	0.83 (0.50, 1.39)	179/931	1.00 (Reference)	29/143	0.99 (0.62, 1.60)
Visual acuity								
URE								
Sex								
Male	158/1455	1.00 (Reference)	27/205	1.65 (0.89, 3.05)	150/1391	1.00 (Reference)	35/269	1.32 (0.76, 2.27)
Female	199/1651	1.00 (Reference)	9/74	1.28 (0.44, 3.76)	189/1567	1.00 (Reference)	19/158	0.93 (0.43, 2.01)
Age								
12–13	188/1587	1.00 (Reference)	20/160	1.66 (0.90, 3.04)	177/1519	1.00 (Reference)	31/228	1.16 (0.72, 1.87)
14–15	169/1519	1.00 (Reference)	16/119	1.23 (0.56, 2.71)	162/1439	1.00 (Reference)	23/199	1.13 (0.65, 1.95)
Race/ethnicity								
Hispanic	168/1267	1.00 (Reference)	9/58	1.61 (0.67, 3.86)	159/1186	1.00 (Reference)	18/139	1.41 (0.66, 2.99)
Non-Hispanic White/Others	63/855	1.00 (Reference)	15/131	1.68 (0.96, 2.93)	66/841	1.00 (Reference)	12/145	1.09 (0.61, 1.95)
Non-Hispanic Black	126/984	1.00 (Reference)	12/90	1.08 (0.49, 2.41)	114/931	1.00 (Reference)	24/143	1.39 (0.84, 2.32)
VI								
Sex								
Male	8/1455	1.00 (Reference)	2/205	2.79 (0.24, 31.93)	6/1391	1.00 (Reference)	4/269	8.48 (1.03, 69.96)^a^
Female	20/1651	1.00 (Reference)	2/74	0.89 (0.13, 6.19)	19/1567	1.00 (Reference)	3/158	0.58 (0.12, 2.74)
Age								
12–13	16/1587	1.00 (Reference)	1/160	0.64 (0.12, 3.40)	14/1519	1.00 (Reference)	3/228	1.00 (0.27, 3.75)
14–15	12/1519	1.00 (Reference)	3/119	4.12 (0.63, 26.86)	11/1439	1.00 (Reference)	4/199	6.43 (1.43, 28.84)^a^
Race/ethnicity								
Hispanic	13/1267	1.00 (Reference)	0/58	NA	10/1186	1.00 (Reference)	3/139	3.37 (0.66, 16.51)
Non-Hispanic White/Others	6/855	1.00 (Reference)	2/131	1.53 (0.21, 10.99)	5/841	1.00 (Reference)	3/145	2.66 (0.54, 12.99)
Non-Hispanic Black	9/984	1.00 (Reference)	2/90	4.19 (0.36, 48.85)	10/931	1.00 (Reference)	1/143	1.01 (0.15, 7.05)

NHANES, National Health and Nutrition Examination Survey; OR, odds ratio; URE, uncorrected refractive error; VI, vision impairment; NA, not applicable.

^aP < 0.05.^

All sample size have been adjusted for age, sex, race/ethnicity, ratio of family income to federal poverty threshold and BMI.

## 4 Discussion

The present study examined the association of myopia, hyperopia, astigmatism, and visual acuity with ADHD and LD, based on a large, representative sample in the US. There is a positive association of hyperopia with ADHD and LD, astigmatism with LD among the children and adolescents in the US. Even after full adjustment, the association remained significant. No significant association was observed between myopia, uncorrected refractive error, visual impairment, and ADHD or LD.

Our results were generally in line with other studies, which also demonstrated an association between hyperopia and ADHD (22). The ability to see is a critical sensory function that is required for information acquisition. Refractive errors, such as astigmatism and hyperopia, can impair a person’s vision and focus, resulting in signs of hyperactivity and inattention. According to studies, a child who has hyperopia may experience headaches, eyestrain, intermittent blur, trouble concentrating on close objects, and poor reading and academic performance due to the need for extra accommodative effort (37, 38).

ADHD and ocular abnormalities are likely interlinked through multiple mechanisms. First, neurodevelopmental dysfuntions during crucial phases of brain growth can affect neural circuits associated with both attention and visual processing. For example, changes in the dopaminergic and noradrenergic systems, which play a vital role in regulating attention and visual perception, have been identified in ADHD. Imbalances in these neurotransmitters may affect the visual cortex and associated visual pathways, potentially leading to visual impairments (39, 40). Some studies have attempted to link alterations in dopaminergic neurotransmitter systems to neuropsychological deficits associated with ADHD. For instance, working memory and attention problems in ADHD may be explained by decreased dopaminergic inputs to the prefrontal cortex (41). Additionally, links have been found between variations of a dopamine receptor gene and sustained attention in ADHD (42). It is commonly recognized that components of human vision as well as higher cognitive abilities depend on an intact dopaminergic neurotransmitter system (43). Secondly, brain regions such as the prefrontal cortex, parietal cortex, and basal ganglia, involved in attentional control and visual information processing, frequently exhibit structural and functional abnormalities in Reduced connectivity between the prefrontal cortex and visual in ADHD. As reported in individuals those with ADHD, reduced connectivity between the prefrontal cortex and visual areas can impede the integration and interpretation of visual stimuli, thereby leading to visual abnormalities. Furthermore, genetic factors and environmental risk factors associated with ADHD may also influence the development of the visual system. The same genetic factors indicated a common biological foundation for the co-existence of these conditions. There was established evidence regarding the role of environmental risk factors in the etiology of ADHD and LD (44), and biological factors in the environment, such as preterm birth (21, 45), systemic infections (46, 47), and prematurity (48, 49) are known to influence vision problems. It is possible that affected neurodevelopment, influenced by these factors, could simultaneously trigger the onset of vision disorders and the symptoms of LD and ADHD. These multiple mechanisms together contribute to the complex relationship between ADHD and visual abnormalities.

Despite finding an association between hyperopia and ADHD or LD, we did not detect a relationship between myopia or visual acuity, which is inconsistent with other findings (50, 51). This is probably because astigmatism and hyperopia affect both far and near vision. By contrast, myopia preserves sharp close vision while only impairing far vision. Due to the increased need for focusing on close objects, astigmatism and hyperopia may become more noticeable in the home or school setting. Consequently, it’s possible that astigmatism and hyperopia have a greater impact on an individual’s ability to focus than myopia (29).

A strength of this research is that the NHANES is a large national sample that was designed to be representative of non-institutionalized children in the US, and thus the results can be generalized. However, our study also has several limitations that should be recognized. First, this is a cross-sectional observational study. As such, we are unable to infer the causal relationship between ADHD and LD and visual impairment in adolescents. Second, the diagnoses of ADHD and LD were based on self-reported information, which might be prone to recall bias. Moreover, our study did not differentiate between specific subtypes of ADHD, such as inattentive (ADHD-I), hyperactive and impulsive (ADHD-H), and combined (ADHD-C) types. Moreover, it lacked qualitative information regarding the specific forms and severity of refractive error and ADHD/LD. Furthermore, we use an older dataset, changes may have occurred in various aspects that could potentially affect the relationship between ADHD and LD and refractive errors. For example, lifestyle changes, such as increased screen time, changes in educational methods, and enhancements in eye-care awareness, may have affected the prevalence and characteristics of ADHD, LD and refractive errors. Furthermore, medication treatment may confound the relationship between visual measurements and ADHD. ADHD drugs that contain stimulants or non-stimulants may have an affect the autonomic nervous system’s regulation of ocular structures (43, 52, 53). Some studies have reported ocular side - effects like accommodation dysfunction, cataracts, mydriasis, and increased intraocular pressure (54–56), while others found no relationship between intraocular pressure and medication treatment (57, 58).

Therefore, more convincing evidence will be required from the prospective cohort studies. Future research should conduct accurate medical examinations to confirm the diagnoses of ADHD and LD, Additionally, it should explore the influence of a broader range of clinical factors on the relationship between ADHD/LD and ocular abnormalities.

## 5 Conclusion

In summary, our research suggests a potential association that children and adolescents diagnosed with ADHD and LD may be at a greater risk of developing hyperopia and astigmatism, while myopia and uncorrected refractive error appear to show no significant association. To accurately diagnose and treat children affected by ADHD and LD, healthcare practitioners from various medical specialties should take this association into account.

## Data Availability

The datasets used in this study can be found at: https://wwwn.cdc.gov/nchs/nhanes/Default.aspx.
